# Risk of infection in roxadustat treatment for anemia in patients with chronic kidney disease: A systematic review with meta-analysis and trial sequential analysis

**DOI:** 10.3389/fphar.2022.967532

**Published:** 2022-09-16

**Authors:** Shan Chong, Qiufen Xie, Tiantian Ma, Qian Xiang, Ying Zhou, Yimin Cui

**Affiliations:** ^1^ Department of Pharmacy, Peking University First Hospital, Beijing, China; ^2^ School of Basic Medicine and Clinical Pharmacy, China Pharmaceutical University, Nanjing, China; ^3^ Department of Nephrology, Peking University First Hospital, Beijing, China

**Keywords:** roxadustat, anemia, chronic kidney disease, meta-analysis, trial sequential analysis

## Abstract

**Background:** Many studies demonstrated that roxadustat (FG-4592) could increase hemoglobin (Hb) levels effectively in anemia patients with chronic kidney disease (CKD). However, its safety remains controversial. This study aims to explore the risk of infection for CKD patients treated with roxadustat, especially focused on sepsis.

**Methods:** We thoroughly searched for the randomized controlled trials (RCTs) comparing treatment with roxadustat versus erythropoiesis stimulating agents (ESAs) or placebo in PubMed, Embase, Cochrane Library, ClinicalTrials.gov, European Union Clinical Trials Register. Both on and not on dialysis anemia patients with CKD were included. Primary outcomes contained the incidence rates of sepsis. Secondary outcomes included infection-related consequences (septic shock and other infection events), general safety outcomes [all-cause mortality, treatment-emergent adverse events (TEAEs) and treatment-emergent serious adverse events (TESAEs)] and iron parameters. Moreover, a trial sequential analysis (TSA) was conducted to assess if the results were supposed to be a robust conclusion.

**Results:** Eighteen RCTs (*n* = 11,305) were included. Overall, the incidence of sepsis (RR: 2.42, 95% CI [1.50, 3.89], *p* = 0.0003) and cellulitis (RR: 2.07, 95% CI [1.24, 3.44], *p* = 0.005) were increased in the roxadustat group compared with placebo group. In non-dialysis-dependent (NDD) CKD patients, the incidence of cellulitis (RR 2.01, 95% CI [1.23, 3.28], *p* = 0.005) was significantly higher in roxadustat group than that in the ESAs or placebo group. Both groups showed similar results in the incidence of septic shock (RR 1.29, 95% CI [0.86, 1.94], *p* = 0.22). A significant increased risk of all-cause mortality [risk ratios (RR): 1.15, 95% confidence interval (CI) [1.05, 1.26], *p* = 0.002] was found in roxadustat treatment, and TSA confirmed the result. Compared with ESAs or placebo, both the incident rates of TEAEs (RR:1.03, 95% CI [1.01, 1.04], *p* = 0.008) and TESAEs (RR: 1.06, 95% CI [1.02, 1.11], *p* = 0.002) were significantly increased in roxadustat group. As for iron parameters, changes from baseline (Δ) of hepcidin (MD: -26.46, 95% CI [-39.83, -13.09], *p* = 0.0001), Δ ferritin and Δ TSAT were remarkably lower in the roxadustat group, while Δ Hb, Δ iron and Δ TIBC increased significantly versus those in ESAs or placebo group.

**Conclusion:** We found evidence that incidence rates of sepsis and cellulitis are higher in roxadustat group compared with placebo. This may be the result of improved iron homeostasis. The risk of all-cause mortality, TEAEs and TESAEs in CKD patients also increased in patients treated with roxadustat. We need more clinical and mechanistic studies to confirm whether roxadustat really causes infection.

## Introduction

Anemia is one of the most common complications of chronic kidney disease (CKD). The prevalence of anemia increased with CKD stage, 17.4%, 50.3%, and 53.4% in stage 3, 4, and 5, respectively (Stauffer and Fan, 2014). Low hemoglobin (Hb) levels cause tachycardia, fatigue, shortness of breath, and even organ damage. Studies have shown that anemia is associated with poor health-related quality of life, increased risk of cardiovascular events, and all-cause mortality (Hanna et al., 2021). For decades, pharmaceutical methods in anemia patients with CKD relies on erythropoiesis stimulating agents (ESAs), iron replacement, and blood transfusions. However, these procedures are associated with severe adverse cardiovascular events and other potential long-term risks (Kliger et al., 2013). Therefore, seeking benefit-risk balance for anemia patients with CKD has become an important issue.

Hypoxia-inducible factors (HIFs) are heterodimeric transcription factors that stimulate erythropoietin (EPO) secretion, and they can be degraded by oxygen-dependent prolyl hydroxylase (PHD) enzymes at a high rate under normoxic conditions. (Joharapurkar et al., 2018). Hypoxia-inducible factors prolyl hydroxylase inhibitor (HIF-PHI) reduce the activity of PHD, resulting in HIF-α accumulation and upregulation of the heterodimer of HIF-α and HIF-β, which stimulate EPO transcription and increase the expression of multiple genes involved in iron metabolism and remedy anemia symptoms (Sanghani and Haase, 2019). These small molecule oral agents thought to be more convenient and safer than ESAs.

Roxadustat is a first-in-class HIF-PHI, which efficacy has been proved in previous studies. It has been approved for treatment of anemia in non-dialysis (NDD) and dialysis (DD) patients in China, and for NDD-CKD patients in Japan due to its correcting and maintaining Hb levels. (Dhillon, 2019). However, the U.S. Food and drug Administration (FDA) refused to approve the marketing application of roxadustat due to safety issues in 2021, and it reported the unexpected signal of severe infection for the first time. (US Food & Drug Administration Department of Health and Human Services, 2021). This report showed a significantly increased incidence of sepsis/septic shock in patients treated with roxadustat, with serious urinary tract infections, cellulitis and peritonitis reinforcing concerns.

Currently, there is a lack of biologic mechanisms, and previous meta-analyses rarely focused on infection-related adverse events. Therefore, it is hard to judge whether this observation comes from a design flaw in a specific study or from roxadustat itself. Patients with CKD are alterations in primary host defense mechanisms and they are 3–4 times more likely to develop infections than the general population (Kidney and Kidney, 2013). Considering the serious health consequences of infection, we conducted this systematic review with meta-analysis and trial sequential analysis (TSA) to summarize the relationship between roxadustat and infection signals in existing clinical studies.

## Methods

This meta-analysis was performed by the guidelines of the Preferred Reporting Items for Systematic Reviews and Meta-Analyses (PRISMA) and was registered prospectively (Registration number: CRD42022299359).

### Search study

PubMed, Embase, Cochrane Library, ClinicalTrials.gov, European Union Clinical Trials Register were searched up to June 2022 for English language studies with the following terms: “roxadustat,” “FG-4592,” “anemia,” “chronic kidney disease,” and “randomized controlled trial” (Supplementary Table S1).

### Selection criteria

Randomized controlled trials (RCTs) were included in the analysis if they met the following criteria: (1) population: CKD patients with anemia with or without dialysis; (2) intervention: roxadustat; (3) control group: ESAs or placebo. Two researchers searched and selected the literature independently. We excluded studies that did not have access to analyzable data. All conflicts are resolved by consensus.

### Data extraction and outcome measures

Full texts and supplementary files of identified studies were reviewed carefully by two researchers independently. The following data were extracted from each study using a predesigned form: basic information of studies (publication year, name of the first author, registration number), characteristics of studies (located, number of patients, phase of trial, study design, patient type, comparator, the initial dose of roxadustat, duration), and safety outcomes.

The primary outcome was the incidence rate of sepsis. The secondary outcomes included infection-related consequences (septic shock, cellulitis, urinary tract infection, peritonitis, pneumonia, device-related infection, nasopharyngitis, osteomyelitis, bronchitis, upper respiratory tract infection, influenza, gastroenteritis, and cystitis), general safety outcomes [all-cause mortality, treatment-emergent adverse events (TEAEs) and treatment-emergent serious adverse events (TESAEs)] and iron parameters [changes from baseline in Hb(Δ Hb), hepcidin (Δ hepcidin), ferritin (Δ ferritin), iron (Δ iron), transferrin saturation (ΔTSAT) and total iron binding capacity (ΔTIBC)].

### Quality Assessment and Statistical analysis

The risk of bias was performed by the Cochrane Collaboration’s tool (Sterne et al., 2019). Each eligible trial was estimated by the six types of bias: selection bias, performance bias, detection bias, attrition bias, reporting bias and other bias. Two researchers complete assessments independently and over the difference by discussion.

The Review Manager (RevMan) 5.4 software was used for meta-analysis and forest plots. For dichotomous outcomes, risk ratio (RR) and 95% confidence interval (CI) were calculated to assess the effect size. For continuous outcomes, mean difference (MD) and 95% CI were pooled. Heterogeneity across studies was performed by *I* ^*2*^ values. *I* ^*2*^ < 50% and ≥50% indicate mild and high heterogeneity, respectively. The fixed-effect model was applied at mild heterogeneity, whereas the random-effect model was utilized at high heterogeneity. Subgroup analyses would be used to explore the underlying causes. Moreover, we conducted sensitivity analysis of the effect of each study to evaluate the stability of the results. Potential publication biases were assessed by Egger’s text, Begg’s text, and funnel plot using R language 4.1.2. All statistical significance level was set at *p* < 0.05 with two-sided.

TSA was incorporated to adjust the random error risk or type Ⅱ error (lack of statistical power). The type Ⅰ error = 5% and power (1-β) = 80%. We calculated the incidence in the control arm and estimated the relative risk reduction by low bias-based. Trial Sequential Analysis Viewer 0.9.5.10 Beta was used to calculated the required information size (RIS).

## Results

### Literature selection

**FIGURE 1:**
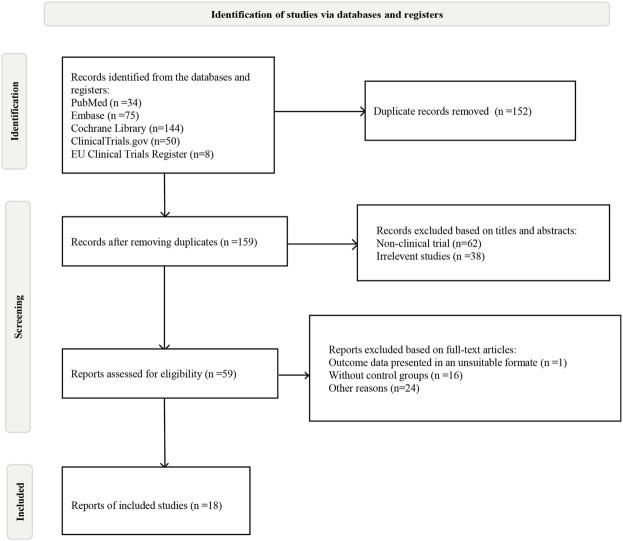
Flow chart of eligible studies.

Initially, 311 articles were obtained based on the search strategy. A total of 152 studies were removed by duplicated. After screening titles and abstracts, 59 studies were left. Finally, 18 eligible RCTs, including 11,305 patients, were included in the meta-analysis (Figure 1).

### Study characteristics

**FIGURE 2:**
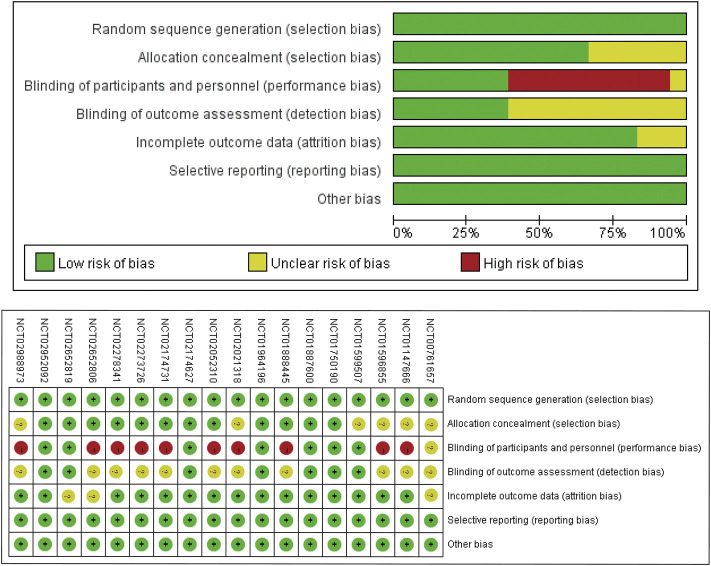
Risk of bias summary. Abbreviations: NCT, National Clinical Trials.

The basic characteristics are showed in Table 1. Of nine RCTs conducted in NDD-CKD patients, seven studies were placebo-controlled, and two studies were ESAs-controlled. All the nine RCTs performed in DD-CKD patients were ESAs-controlled. We used Cochrane Collaboration’s tool to assess the risk of bias for included studies. Most RCTs were of acceptable quality. The open-label designed RCTs had high risk of blinding biases. The unclear information of random sequence generation and allocation concealment provided major sources of selection biases (Figure 2).

**TABLE 1:** Basic characteristics of included studies.

Study	Registration number	Located	No. of patients	Phase of trial	Study design	Patients type	Comparator	Initial dose of roxadustat	Duration (weeks)
Besarab 2015 Besarab et al. (2015)	NCT00761657	US	116	2	Randomized, single-blind, multicenter	NDD	Placebo	0.7, 1, 1.5, 2.0 mg/kg; BIW or TIW	4
Akizawa 2020 Akizawa et al. (2020)	NCT02952092	JP	301	2	Randomized, double-blind, multicenter	DD	ESAs	70, 100mg; TIW	24
Provenzano 2016 Provenzano et al. (2016)	NCT01147666	US	144	2	Randomized, open-label, multicenter	DD	ESAs	1.0, 1.5, 1.8, 2.0 mg/kg; TIW	6, 19
Akizawa 2019 Akizawa et al. (2019)	NCT01964196	JP	107	2	Randomized, double-blind, multicenter	NDD	Placebo	50, 70, 100mg; TIW	24
Chen2017(NDD) Chen et al. (2017)	NCT01599507	CHN	91	2	Randomized, double-blind, multicenter	NDD	Placebo	1.1–1.75 mg/kg, 1.50–2.25 mg/kg; TIW	8
Chen2017 (DD) Chen et al. (2017)	NCT01596855	CHN	96	2	Randomized, open-label, multicenter	DD	ESAs	1.1–1.8, 1.5–2.3, 1.7–2.3 mg/kg; TIW	6
Provenzano 2021 Provenzano et al. (2021)	NCT02052310	Muti.	1039	3	Randomized, open-label, multicenter	DD	ESAs	70, 100mg; TIW	52
Shutov 2021 Shutov et al. (2021)	NCT01887600	Muti.	594	3	Randomized, double-blind, multicenter	NDD	Placebo	70, 100mg; TIW	52
Fishbane2021(NDD) Fishbane et al. (2021)	NCT02174627	Muti.	2761	3	Randomized, double-blind, multicenter	NDD	Placebo	70mg; TIW	52
Barratt 2021 Barratt et al. (2021a)	NCT02021318	Muti.	616	3	Randomized, open-label, multicenter	NDD	ESAs	70, 100mg; TIW	104
Csiky 2021 Csiky et al. (2021)	NCT02278341	Muti.	834	3	Randomized, open-label, multicenter	DD	ESAs	70–200mg; TIW	52
Akizawa 2021 Akizawa et al. (2021)	NCT02988973	JP	262	3	Randomized, open-label, multicenter	NDD	ESAs	70, 100mg; TIW	52
Charytan 2021 Charytan et al. (2021)	NCT02273726	US	741	3	Randomized, open-label, multicenter	DD	ESAs	50, 70, 100, 200mg; TIW	52
Chen2019 (DD) Chen et al. (2019a)	NCT02652806	CHN	304	3	Randomized, open-label, multicenter	DD	ESAs	100, 120mg; TIW	26
Chen2019(NDD) Chen et al. (2019b)	NCT02652819	CHN	152	3	Randomized, double-blind, multicenter	NDD	Placebo	70, 100mg; TIW	8
Coyne 2020 Coyne et al. (2020)	NCT01750190	Muti.	916	3	Randomized, double-blind, multicenter	NDD	Placebo	70, 100mg; TIW	52
Fishbane2021 (DD) Fishbane et al. (2022)	NCT02174731	Muti.	2101	3	Randomized, open-label, multicenter	DD	ESAs	70, 100mg; TIW	52
Astellas 2018 Astellas Pharma Inc (2020)	NCT01888445	JP	130	2	Randomized, open-label, multicenter	DD	ESAs	50, 70, 100mg; TIW	28

Abbreviations: CHN, China; JP, Japan; US, The United States; Muti., Multiple countries. DD, dialysis- dependent patients; NDD, non-dialysis-dependent patients. QW, once a week; BIW, twice weekly; TIW, three times weekly.

### Outcomes

**FIGURE 3:**
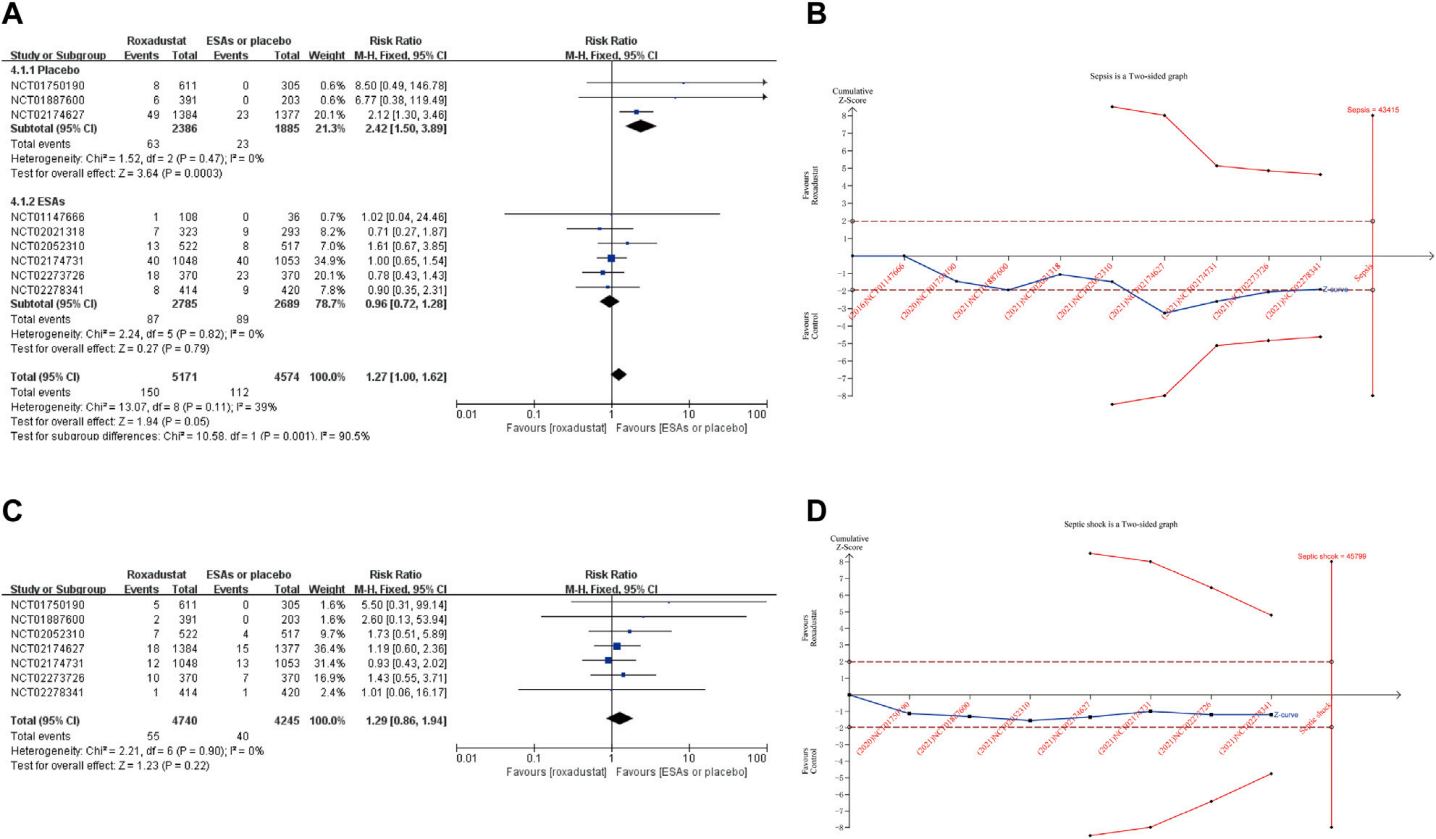
Forest plot of sepsis **(A)** and septic shock **(C)**. TSA of sepsis **(B)** and septic shock **(D)**. Abbreviations: ESAs, erythropoiesis stimulating agents.

The incidence rate of sepsis is the primary outcome of this meta-analysis. Nine studies reported this data. Pool results of included studies with fixed-effects model showed no significant difference in the incidence rates of sepsis between roxadustat group and ESAs or placebo group (RR: 1.27, 95% CI [1.00,1.62], *p* = 0.05) (Figure 3A). However, after kicking out the highest weight study (NCT02174731, weight 34.9%), the conclusion became significant (RR: 1.41, 95%CI [1.05,1.90], *p* = 0.02) (Supplementary Figure S1). Further TSA demonstrated that the Z-curve of sepsis neither crossed the O’ Brien-Fleming monitoring boundary nor entered the RIS (*n* = 43,415). This result showed that we need more studies to confirm the result (Figure 3B). Subgroup analysis demonstrated a significantly higher incidence of sepsis in the roxadustat group than in the placebo group (RR: 2.42, 95% CI [1.50, 3.89], *p* = 0.0003).

The incidence rate of septic shock was reported in 7 studies, and our results showed a similar risk between roxadustat and the use of placebo or ESAs (RR: 1.29, 95% CI [0.86,1.94], *p* = 0.22) (Figure 3C) Likewise, TSA did not confirm the result (Figure 3D). Incidence rates of cellulitis in the roxadustat group were similar to that in the ESAs or placebo groups (RR: 1.29, 95% CI [0.93, 1.80], *p* = 0.13). And subgroup analysis confirmed that roxadustat treatment significantly increased incidence rates of cellulitis in the NDD-CKD (RR: 2.01, 95% CI [1.23, 3.28], *p* = 0.005) and placebo-controlled studies (RR: 2.07, 95% CI [1.24, 3.44], *p* = 0.005). Patients on roxadustat treatment had a remarkable higher incidence of urinary tract infection than those on placebo or ESAs (RR: 1.35, 95% CI [1.14, 1.60], *p* = 0.0005). After removing the highest weight study (NCT02174627), the result was no longer reliable (RR: 1.08, 95% CI [0.84, 1.41], *p* = 0.54) (Supplementary Figure S1). Compared with ESAs or placebo, roxadustat did not increase the risk of peritonitis (RR: 1.23, 95% CI [0.88, 1.72], *p* = 0.23), pneumonia (RR:1.05, 95% CI [0.92, 1.20] *p* = 0.49), device-related infection (RR: 1.38, 95% CI [0.88, 2.17], *p* = 0.16), nasopharyngitis (RR: 1.02, 95% CI [0.82, 1.27], *p* = 0.84), osteomyelitis (RR: 0.84, 95% CI [0.51, 1.40], *p* = 0.51), bronchitis (RR: 0.97, 95% CI [0.82, 1.16], *p* = 0.76), upper respiratory tract infection (RR: 1.14, 95% CI [0.99, 1.31], *p* = 0.08), influenza (RR: 1.05, 95% CI [0.59, 1.87], *p* = 0.88), gastroenteritis (RR: 1.25, 95% CI [0.81, 1.94], *p* = 0.31) and cystitis (RR: 1.62, 95% CI [0.68, 3.83], *p* = 0.75) (Supplementary Figure S2).

**FIGURE 4:**
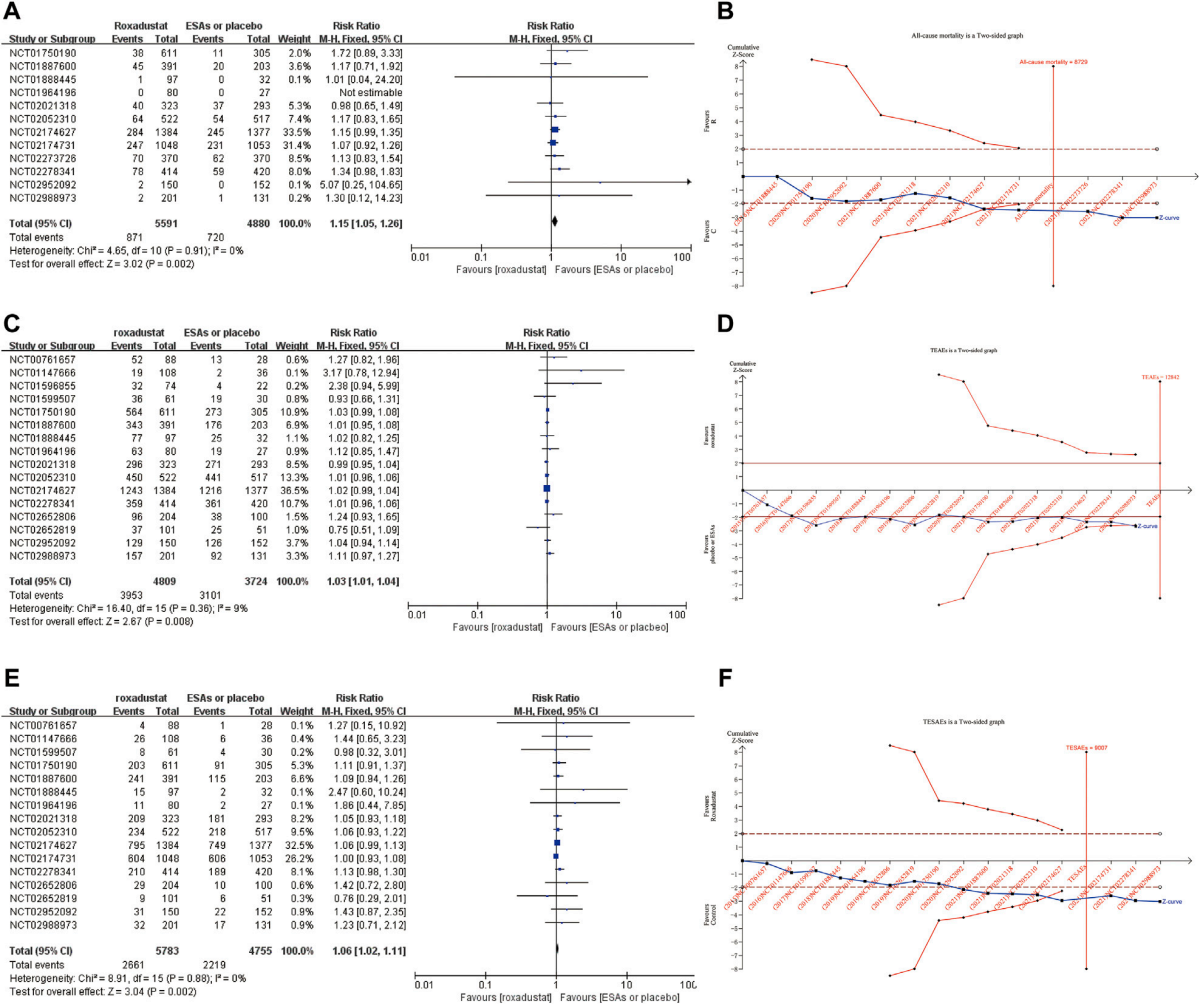
Forest plot of all-cause mortality **(A)**, TEAEs **(C)**, and TESAEs **(E)**. TSA of all-cause mortality **(B)**, TEAEs **(D)**, and TESAEs **(F)**. Abbreviations: ESAs, erythropoiesis stimulating agents.

Incidence rates of all-cause mortality was reported in 12 included studies. The pooled results using a fix-effects model illustrated that the incidence of all-cause mortality was significantly higher in the roxadustat group than in ESAs or placebo group (RR:1.15, 95% CI [1.05, 1.26], *p* = 0.002) (Figure 4A). TSA was conducted to confirm this result (Figure 4B). The incidence rate of all-cause mortality has crossed its trial sequential monitoring boundary (RIS = 8729, actual sample size 10,471), the cumulative Z-curve had crossed the trial sequential monitoring boundary, which meant the response of roxadustat was truly positive. Further, sixteen studies investigated incidence rates of TEAEs. Roxadustat treated patients experienced significantly more risk of TEAEs (RR:1.03, 95% CI [1.01, 1.04], *p* = 0.008) compared with ESAs or placebo, and TSA confirmed the result (Figures 4C,D). We investigated the incidence rates of TESAEs in 16 studies. The pooled results with a fixed-effect model suggested patients treated with roxadustat were more likely to experience TESAEs (RR: 1.06, 95% CI [1.02, 1.11], *p* = 0.002), and TSA also confirmed this result (Figures 4E,F).

Iron paraments were conducted in this meta-analysis. Δ Hb, the core outcome of anemia therapy, raised significantly in roxadustat group compared with ESAs or placebo (MD: 0.86, 95% CI [0.47, 1.25], *p* < 0.0001). The commonly used parameters of iron metabolism are hepcidin, ferritin, iron, TSAT, and TIBC. On the one hand, the pooled results displayed with the random-effects model showed that Δ hepcidin (MD: −26.46, 95% CI [−39.83, −13.09], *p* = 0.0001), Δ ferritin (MD: −39.81, 95% CI [−63.52, −16.11], *p* < 0.0010), Δ TSAT (MD: −1.76, 95% CI [−3.96, 0.43], *p* < 0.00001) were lower in the roxadustat group. On the other hand, Δ iron (MD: 2.76, 95% CI [0.97, 4.56], *p* = 0.003) and Δ TIBC (MD: 53.44, 95% CI [41.14, 65.74], *p* < 0.00001) increased in patients treated with roxadustat (Supplementary Figure S2). Furthermore, subgroup analysis obtained that Δ hepcidin (MD: −32.65, 95% CI [−43.24, -22.06], *p* < 0.00001) and Δ ferritin (MD: −52.27, 95% CI [−62.97, −41.58], *p* < 0.00001) decreased significantly in roxadustat group for NDD-CKD patients, whereas results were similar in DD-CKD patients (Supplementary Table S2).

### Publication bias and sensitivity analysis

The result of Egger’s and Begg’s test did not show the evidence of publication bias except TEAEs (*p*_Begg’s_ = 0.04), hepcidin (*p*_Egger’s_ = 0.00) and TIBC (*p*_Egger’s_ = 0.04) (Supplementary Table S3). Funnel plot of all outcomes was provided in Supplementary Figure S3. In sensitivity analysis, pool results of sepsis and urinary tract infection altered after being removed from the study with the highest weight. The details of sensitivity analysis were shown in Supplementary Figure S1.

## Discussion

Over 20 clinical trials have demonstrated that roxadustat can effectively improve anemia in patients with CKD by increasing Hb levels and regulating iron metabolism, and studies suggested that roxadustat may be available in more pathological conditions (Miao et al., 2022). The lack of real-world safety data is an obstacle to broader acceptance. In this meta-analysis, we performed 17 outcomes to describe the safety in roxadustat treatment. Roxadustat treatment showed a higher risk of sepsis and cellulitis than placebo. Meanwhile, roxadustat had significantly increased the incidence rates of all-cause mortality, TEAEs and TESAEs of CKD patients compared with either ESAs or placebo. Moreover, results of 6 iron parameters supported roxadustat treatment promoted iron utilization.

Non-clinical studies have shown that HIF plays an essential role in sepsis. First, HIF is involved in the process of sepsis via macrophages (Kiss et al., 2012). In the course of sepsis, the expression of HIF changes dynamically, which has a significant impact on cytokine change and clinical symptoms. Therefore, some researchers have proposed HIF as a potential biomarker of sepsis (Fitzpatrick, 2019). A clinical trial (NCT02163473) attempted to take HIF-1 as a new biomarker for septic shock, the results of which have yet to be published. Second, HIF is a potential target for the treatment of sepsis. *In vivo* and *in vitro* experiments (Han et al., 2020) demonstrated that roxadustat could upregulate HO-1 by increasing HIF-1 α level, thereby attenuating Lipopolysaccharide (LPS) -induced inflammation, alleviating lung injury prolong survival of septic mice. An endogenous metabolite of estradiol reduces lung, liver, and kidney injury by inhibiting inducible NO synthase (iNOS)/NO and cytokines through HIF-1α signaling (Yeh et al., 2011). Shalova et al. (2015) propose that the role of HIF-1 α in sepsis is related to the time phase, that is, the activation of human monocyte HIF-1 α promotes the inflammatory phenotype in the initial phase, while HIF-1 α induces the expression of IRAKM to make the cell endotoxin tolerant state and inhibit the inflammatory response in the resolution phase. This conclusion may support the higher incidence of sepsis and similar incidence of septic shock in the roxadustat group compared with ESAs or placebo in the present study.

Relatedness is the most important consideration when assessing the adverse events of drugs. Among the 12 common infection-related adverse events investigated in this study, there was a significant risk difference between the roxadustat group and placebo group in the incidence of cellulitis. Numerically greater risk was found for the roxadustat group than the ESAs or placebo group, including peritonitis, urinary tract infections, nasopharyngitis, pneumonia, device-related infections, upper respiratory tract infections, gastrointestinal infections, influenza, and cystitis. Although the results were not significant or stable. In a pooled analysis of three large RCTs (NCT02174627, NCT01750190, NCT01887600) (US Food & Drug Administration Department of Health and Human Services, 2021), urinary tract infection [risk difference (RD) 0.3, RR based on patients-year (P-Y) 1.13], bacterial infection (RD 0.5, RR based on P-Y 1.73), cellulitis (RD 0.4, RR based on P-Y 1.66) and peritonitis (RD 0.3, RR based on P-Y 1.68) were significantly increased in roxadustat group. In addition, the most commonly TEAEs of Daprodustat, another HIF-PHI, was nasopharyngitis [32% (9 of 28)] and infected dermal cyst (7% [2 of 28]), although there was no clear evidence of causality (Dhillon, 2020). These signals are consistent, suggesting that roxadustat may increase the risk of sepsis in patients with CKD. For subsequent studies, we recommend paying close attention to the clinical details (such as dose, timing, comorbidities, etc.) of patients receiving roxadustat to infer the exact relationship between the drug and adverse events and the influencing factors.

Our study suggests that patients with CKD who take roxadustat are at higher risk of infection. But the reason is not well understood. This meta-analysis supports iron hypotheses. Various iron sources in the human body provide growth-limiting nutrients to potential pathogens. Thus, the host-defense mechanism builts hypoferremia during inflammation. Hepcidin is the master regulator of iron trafficking, inhibiting iron absorption from the intestine and releasing recycled iron from the macrophage (Drakesmith and Prentice, 2012). Hepcidin knockout mice become iron-loaded (Nicolas et al., 2001). On the one hand, our results supported that HIF-PHI enhances iron uptake and mobilization by decreasing hepcidin in anemia patients. On the other hand, elevated iron levels enhance vulnerability to pathogens, such as vibrio species, Nontyphoidal Salmonella and malaria (Nairz and Weiss, 2020). In macrophages, iron loading stimulates tricarboxylic cycle activity via upregulating the expression of aconitase, resulting in promoting aerobic glycolysis and a pathogen-friendly environment. Moreover, a prospective study in sepsis patients demonstrated that elevated serum iron and ferritin increased mortality, and TSAT improved the predictive power of SOFA score for survival in septic intensive care unit patients (Brandtner et al., 2020). There are other hypotheses. Eleftheriadis et al. (2021) supposed that roxadustat might affect the adaptive immune system and increase urinary tract infection and pneumonia incidence. While Liu et al. (2021) indicated that long-term use of roxadustat may lead to the progression of renal cysts and cause adverse events of infection. More studies are needed to confirm that roxadustat predisposes patients to infection or that these results are merely statistical differences.

Compared with ESAs or placebo, roxadustat treated patients showed higher all-cause mortality. Similarly, Kaplan-Meier survival curves from a FDA report (U.S.Food & Drug Administration, 2021) supported that the OT+28 roxadustat has statistically significantly higher mortality than ESAs, although the causes of death were not adjudicated. It is worth mentioning that some previous studies reported no statistical difference in all-cause mortality between the roxadustat group and the ESAs or placebo group due to shorter follow-up (Barratt et al., 2021b) or earlier publication (Tang et al., 2021). Although both studies found slightly higher all-cause mortality in the roxadustat group. Interestingly, the FDA report showed that the number of adjudicated deaths from infection (RD 0.71, RR based on P-Y 2.09) was higher in the roxadustat group compared with placebo in NDD-CKD patients, and sepsis/septic shock (RD 1.3, RR based on P-Y 2.37) of serious adverse events also increased in roxadustat treatment (US Food & Drug Administration Department of Health and Human Services, 2021). However, few details of the death were disclosed. According to the available study (Akizawa et al., 2020; Astellas Pharma Inc, 2020; Akizawa et al., 2021; Csiky et al., 2021; US Food & Drug Administration Department of Health and Human Services, 2021), we speculate that cardiovascular disorder is the leading cause of death, and infections are probably the second or third. Identifying the weight of deaths due to infection is vital for studying the consequences of infection. In addition, the responses to anemia treatment between the dead and the survivor are of concern. These questions deserve further research. Notably, the result of NCT02278341 demonstrated that several risk factors affect death events, including age, prior ESA type/dose, diabetes, medication, baseline low-density lipoproteins, and time since the start of dialysis. Thus, these covariates should be considered in further research. For now, due to the significantly higher incidence of all-cause mortality, TEAEs, TESAEs and sepsis in patients treated with roxadustat, physicians should carefully evaluate before recommending. Patients with infections, blood or lymphatic diseases are not advised for the agent (Zheng et al., 2021).

This meta-analysis has several strengths. Firstly, we focused on infection-related adverse events for roxadustat, which has rarely been mentioned. Secondly, it incorporates the latest nine clinical data completed in 2021 and covers more extended follow-up periods. Thirdly, low heterogeneity exists in all safety outcomes, with *I* ^2^ < 39%. The *p*-values of Egger’s tests for all safety indicators were ≥0.05. These results indicate that our study have high reliability. We also admit that our study has limitations. First, the evidence for this meta-analysis is based on multiple RCTs worldwide from 2015 to 2021. The adverse events were defined differently in different studies, which might increase the risk of outcome biases. Second, strict inclusion and exclusion criteria ensured minimal population interference. It is helpful to compare the relative risk of event occurrence between roxadustat and ESAs or placebo treatment. However, this might underestimate the absolute risk of real-world adverse reactions to roxadustat. Third, TSA showed that the Z-curve of sepsis and septic shock neither crossed the O′ Brien-Fleming monitoring boundary nor entered the RIS. More studies were needed to confirm our results. Fourth, all the included studies were sponsored by pharmaceutical companies. We expect more researcher-sponsored research to emerge in the future.

## Conclusion

In summary, a higher incidence rates of sepsis and cellulitis was found in roxadustat group compared to placebo. This may be the result of improved iron homeostasis. The risk of all-cause mortality, TEAEs and TESAEs in CKD patients were also increased in patients treated with roxadustat. It was appropriate for health providers to pay close attention to the potential risk of infection and make a careful assessment before prescribing roxadustat. Future studies should focus more on details of patients who suffered from infection or death.

## Data Availability

The original contributions presented in the study are included in the article/Supplementary Material; further inquiries can be directed to the corresponding author.
