# Supplementary material for: Risk of infection in roxadustat treatment for anemia in patients with chronic kidney disease: A systematic review with meta-analysis and trial sequential analysis
Source: Front Pharmacol. 2022 Sep 16;13:967532. doi: 10.3389/fphar.2022.967532 (PMC9523222; doi:10.3389/fphar.2022.967532)
Supplement: Supplementary file 1 [file DataSheet1.docx]

Supplementary Material

# Supplementary Figures and Tables

## Supplementary Figures

**
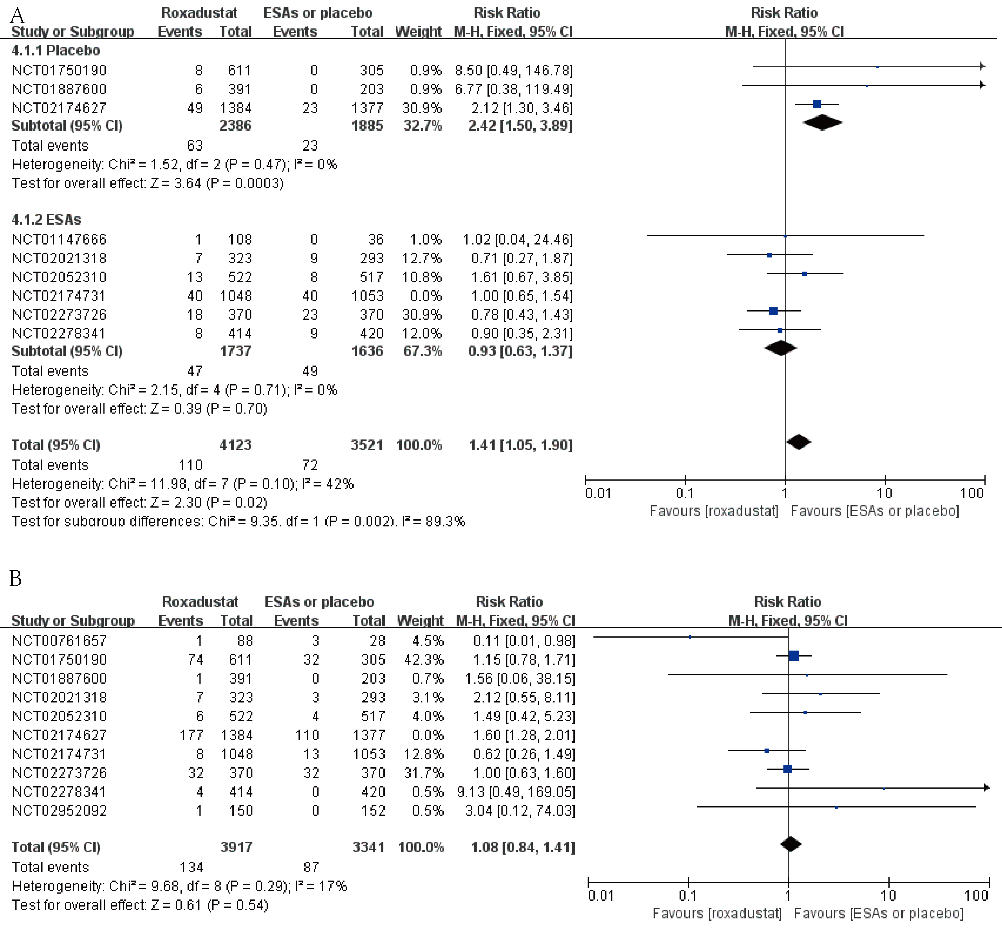
**

**Supplementary Figure S1.** Sensitivity analysis. Sepsis (A) and urinary tract infection(B).

**
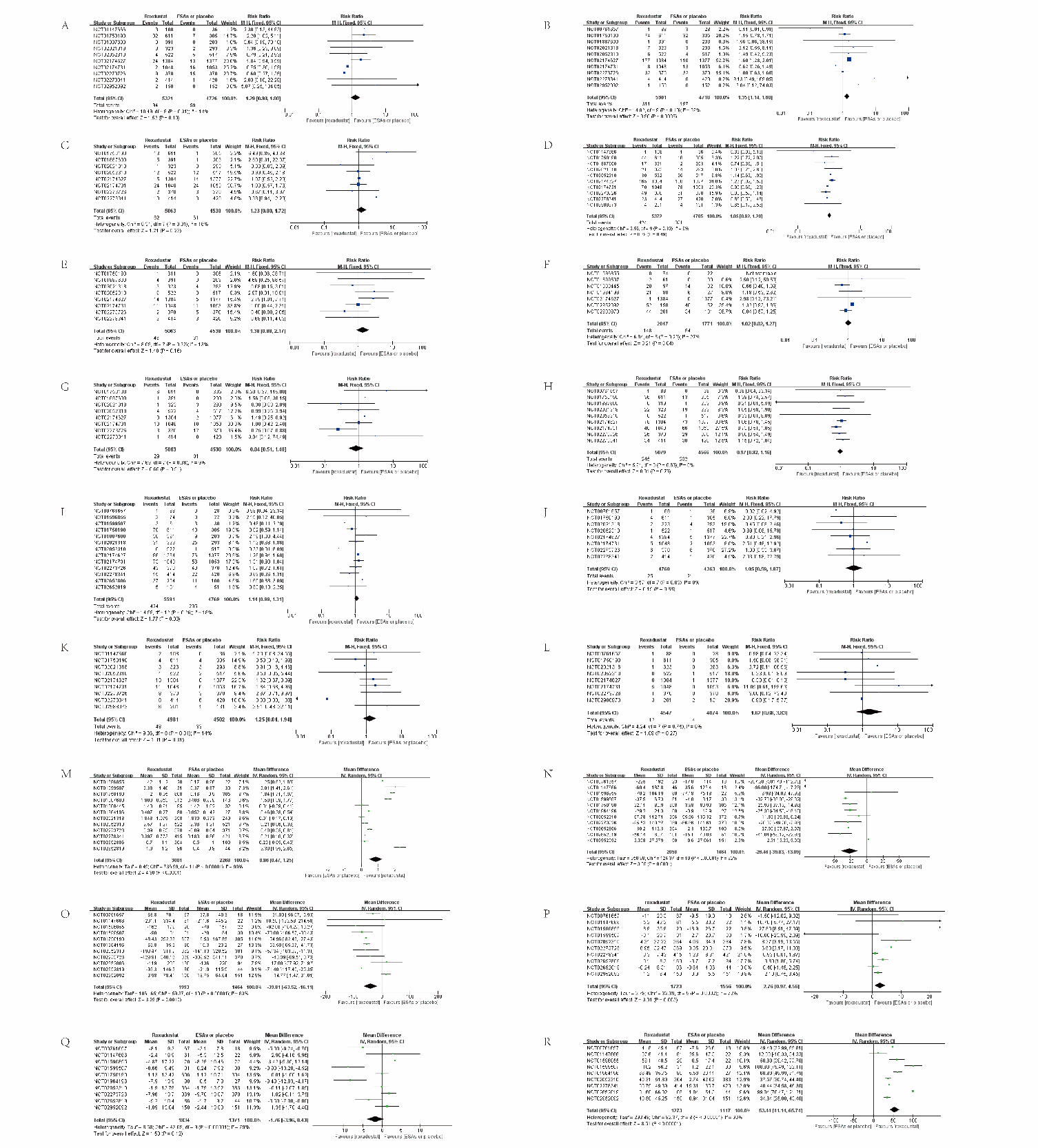
**

**Supplementary Figure S2.** Forest plot of cellulitis(A), urinary tract infection(B), peritonitis(C), pneumonia(D), device-related infection(E), nasopharyngitis(F), osteomyelitis(G), bronchitis(H), upper respiratory tract infection(I), influenza(J), gastroenteritis(K), cystitis(L), ΔHb(M), Δhepcidin(N), Δferritin(O), Δiron(P), ΔTSAT(Q) and ΔTIBC(R).


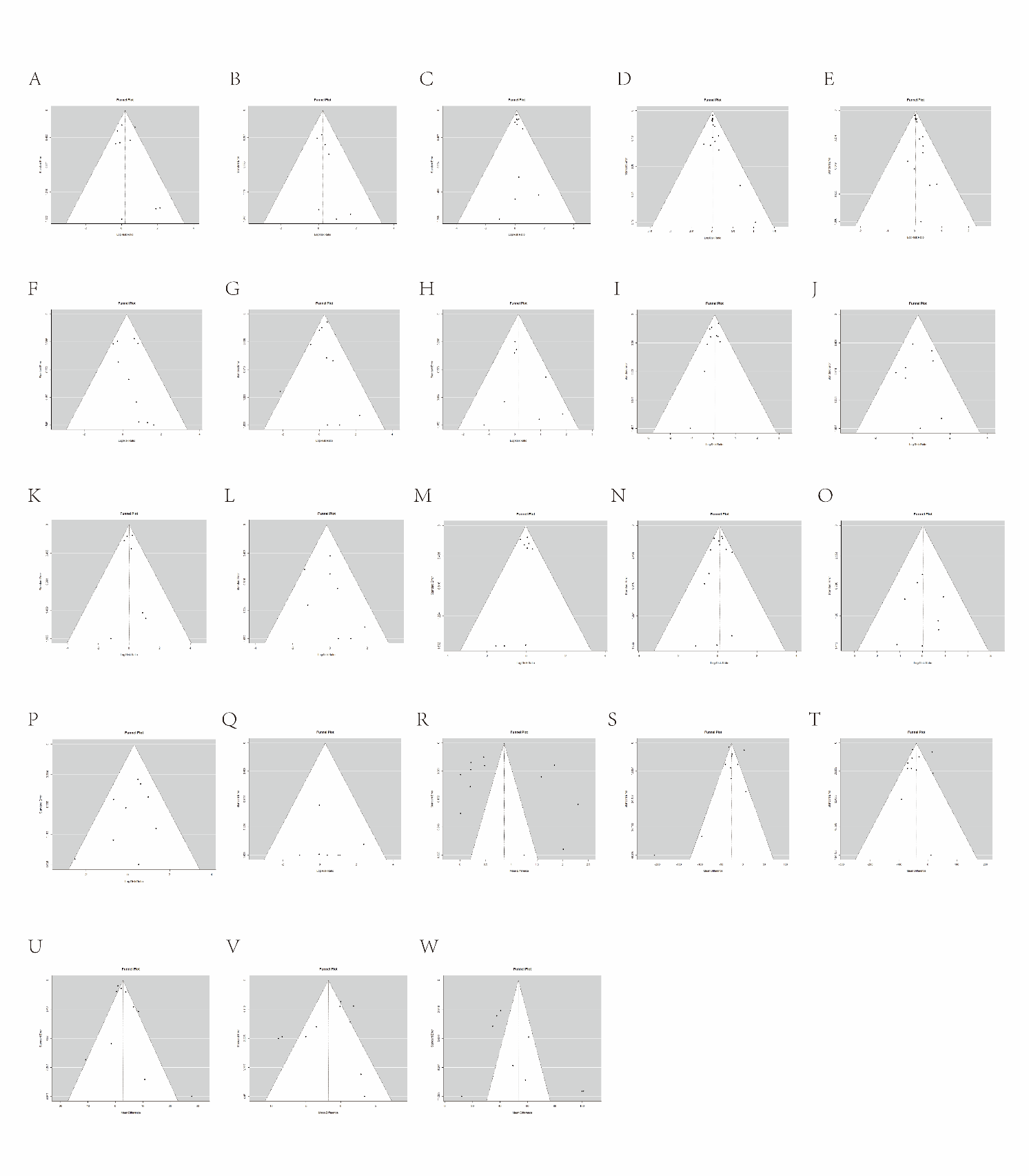


**Supplementary Figure S3.** Funnel plot of sepsis(A), septic shock(B), all-cause mortality(C), TEAEs(D), TESAEs(E), cellulitis(F), urinary tract infection(G), peritonitis(H), pneumonia(I), device-related infection(J), nasopharyngitis(K), osteomyelitis(L), bronchitis(M), upper respiratory tract infection(N), influenza(O), gastroenteritis(P), cystitis(Q), ΔHb(R), Δhepcidin(S), Δferritin(T), Δiron(U), ΔTSAT(V) and ΔTIBC(W).

## Supplementary Tables

**Supplementary Table S1. Searching strategies**

| Database | Searching strategies |
| --- | --- |
| Pubmed | ((("FG-4592" [Supplementary Concept]) OR (roxadustat)) AND ((((Chronic kidney disease) OR (CKD)) OR (anemia)) OR (dialysis))) AND ((("Randomized Controlled Trial" [Publication Type]) OR "Randomized Controlled Trials as Topic"[Mesh])) |
| Embase | ('chronic kidney disease'/de OR 'anemia'/de) AND ('roxadustat'/mj OR 'FG-4592'/mj) AND ('randomized controlled trial'/exp) |
| Cochrane Library | (roxadustat) OR (FG-4592) AND (chronic kidney disease) AND (randomized controlled trials) |
| ClinicalTrials.gov | roxadustat |
| European Union Clinical Trials Register | roxadustat |

**Supplementary Table S2. Subgroup analysis of efficacy outcomes**

| Outcomes | Subgroup | Included trials | MD (95% CI) | p-value |
| --- | --- | --- | --- | --- |
| Δ Hb | DD | 6 | 0.31 (0.14, 0.48) | 0.0005 |
|  | NDD | 6 | 1.35 (0.64, 2.06) | 0.0002 |
| Δ Hepcidin | DD | 6 | -15.18 (-32.69, 2.33) | 0.09 |
|  | NDD | 5 | -32.65 (-43.24, -22.06) | <0.00001 |
| Δ Ferritin | DD | 6 | -20.78 (-58.04, 16.48) | 0.27 |
|  | NDD | 5 | -52.27 (-62.97, -41.58) | <0.00001 |
| Δ Iron | DD | 7 | 3.85 (1.71, 5.98) | 0.0004 |
|  | NDD | 3 | -1.19 (-5.98, 3.61) | 0.63 |
| Δ TSAT | DD | 5 | 1.06 (-0.17, 2.28) | 0.09 |
|  | NDD | 5 | -4.89 (-8.74, -1.03) | 0.01 |
| Δ TIBC | DD | 5 | 37.79 (30.44, 45.13) | <0.00001 |
|  | NDD | 4 | 76.53 (52.70, 100.37) | <0.00001 |

Abbreviations: hemoglobin, Hb; dialysis, DD; non-dialysis, NDD

**Supplementary Table S3. Publication bias**

| **Outcomes** | *p* _Egger’s_ | *p* _Begg’s_ |
| --- | --- | --- |
| Sepsis | 0.4115 | 0.7614 |
| Septic shock | 0.3317 | 0.1361 |
| All-cause mortality | 0.5113 | 0.7373 |
| TEAEs | 0.2028 | 0.0413* |
| TESAEs | 0.1211 | 0.0641 |
| Cellulitis | 0.8365 | 0.3988 |
| Urinary tract infection | 0.5615 | 0.9145 |
| Peritonitis | 0.3558 | 0.9049 |
| Pneumonia | 0.1922 | 0.8618 |
| Device-related infection | 0.9151 | 0.9049 |
| Nasopharyngitis | 0.8561 | 1.0000 |
| Osteomyelitis | 0.3488 | 0.2751 |
| Bronchitis | 0.5741 | 0.6122 |
| Upper respiratory tract infection | 0.8577 | 0.5546 |
| Influenza | 0.9368 | 0.9049 |
| Gastroenteritis | 0.1901 | 0.3585 |
| Cystitis | 0.8365 | 0.3988 |
| Hb | 0.1118 | 0.3807 |
| Hepcidin | 0.0046* | 0.5423 |
| Ferritin | 0.8255 | 0.4454 |
| Iron | 0.1237 | 0.2164 |
| TSAT | 0.6372 | 0.2912 |
| TIBC | 0.0367* | 0.4767 |

*p<0.05

Abbreviations: TEAEs, treatment-emergent adverse events; TESAEs, treatment-emergent serious adverse events; TSAT, transferrin saturation; total iron binding capacity, TIBC
